# A novel phenotype-genotype relationship with a *TGFBI* exon 14 mutation in a pedigree with a unique corneal dystrophy of Bowman’s layer

**Published:** 2008-08-18

**Authors:** Catherine E. Wheeldon, Betina H. de Karolyi, Dipika V. Patel, Trevor Sherwin, Charles N.J. McGhee, Andrea L. Vincent

**Affiliations:** Department of Ophthalmology, Faculty of Medical and Health Sciences, University of Auckland, Auckland, New Zealand

## Abstract

**Purpose:**

Corneal dystrophy of Bowman’s layer (CDB) belongs to a group of dystrophies associated with mutations in the transforming growth factor-beta-induced (*TGFBI*) gene. CDB is further divided into a geographic variant (CDB1/Reis Bücklers, RBCD), and a honeycomb variant (CDB2/Thiel Behnke, TBCD). We undertook mutational analysis of *TGFBI* in a family with an unusual CDB variant and describe a novel phenotype-genotype association.

**Methods:**

Individuals from a pedigree with CDB underwent extensive phenotyping, including laser scanning in vivo confocal microscopy, and histological examination of four corneal buttons obtained at penetrating keratoplasty. Transmission electron microscopy of an excised allograft cornea from one affected individual was also performed. Following informed consent, DNA samples were collected. Polymerase chain reaction (PCR) and sequencing of all coding exons of *TGFBI* was performed. Family members were recruited with subsequent phenotyping and genotyping, and paternity testing.

**Results:**

Clinical examination and other phenotypic information confirmed a diagnosis of CDB, with various features either more suggestive of CDB1 or of CDB2. A mutation in exon 14, H626P, segregated with the disease in this pedigree. This mutation was confirmed with NlaIII restriction enzyme digest, and was not seen in 100 control chromosomes.

**Conclusions:**

Within this pedigree, CDB segregates with an H626P mutation, which is previously described occurring in lattice corneal dystrophy. The majority of mutations in *TGFBI* previously described segregating with CDB1 and CDB2 are R124L and R555Q, respectively. Although a Bowman’s layer dystrophy, the phenotype in this pedigree does not closely conform to the classical diagnostic criteria for either CDB1 or CDB2, and therefore represents a novel phenotype-genotype correlation.

## Introduction

Reis-Bücklers corneal dystrophy (RBCD/CDB1, OMIM 608470) was first described by Reis in 1917 as an annular dystrophy (dystrophia anularis) [1]. Bücklers re-examined the same pedigree in 1949 and showed that the dystrophy was present in four consecutive generations [2], and affected members consistently presented with painful corneal erosions in childhood, with geographic opacities seen at the level of Bowman’s layer. Thiel and Behnke described a similar corneal dystrophy in 1967 that presented with recurrent erosions, moderately reduced vision, autosomal dominant inheritance, and honeycomb shaped opacities at the level of Bowman’s layer (TBCD/CDB2, OMIM 602082) [3].

Since the report of Thiel and Behnke, descriptions in the literature have varied in nomenclature and classification, rendering differentiation between autosomal dominant CDB1 and CDB2 difficult [4-7]. In a comprehensive review preceding genetic characterization, Küchle et al. [5] proposed that these dystrophies are indeed two distinct entities that differ markedly in their appearance at the slit-lamp, associated degree of visual loss, histopathological findings, transmission electron microscopy (TEM) features, and prognosis following corneal transplantation. Using Kuchle’s classification - clinically, CDB1 is described as exhibiting confluent geographic opacities at the level of Bowman’s layer and histopathology demonstrates band shaped granular, subepithelial deposits which stain intensely red with Masson trichrome. TEM reveals the presence of “rod shaped bodies” [5]. In contrast CDB2 has honeycomb shaped opacities, a subepithelial fibrocellular layer interposed between the epithelium and stroma, stains less strongly with Masson trichrome and has “curly collagen” fibers present on TEM [5]. While visual loss with CDB1 is described as early and marked [1,2,5], with CDB2 it is later and moderate [3,5]. The recurrence of the dystrophy after corneal transplantation is noted to be early with CDB1 and much later with CDB2 [5].

Following linkage of CDB1 and other corneal dystrophies (Granular, Avellino, and Lattice) to 5q31 [8,9], Munier et al. [10] reported mutations in the *TGFBI* (transforming growth factor -beta-induced) gene (OMIM 601692) correlating the R555Q mutation with CDB1. However, later, clinical, molecular and ultrastructural findings have demonstrated that it is actually CDB2 that correlates with the mutation R555Q and CDB1 to the mutation R124L [11-14].

*TGFBI*, initially called *BIGH3* [15], encodes for a 683 amino acid protein product, with several nomenclatures including “keratoepithelin” and the now preferred “TGFBI associated protein,” TGFBIp. This extracellular matrix protein is expressed in many tissues including the corneal epithelium [16], and contains four regions of internal homology known as Fasciclin-like (Fas) domains. The protein has high sequence homology to fasciclin1, an insect cell adhesion molecule, and is thought to play a role in cell adhesion [17]. The majority of mutations in *TGFBI* reported to date have been located either within the codon encoding for amino acid R124, or at the boundary of, or within the Fas domain 4 [4,12], although a few mutations outside these regions are also reported [18-22]. Immunohistological studies have shown TGFBIp to be present in the abnormal corneal deposits seen in certain corneal dystrophies [23], as well as in secondary amyloidosis in corneal disease unrelated to *TGFBI* mutations [24].

We report a four-generation family with an autosomal dominant dystrophy that was initially diagnosed as CDB1 on the basis of clinical appearance and clinical course. Comprehensive characterization of the disease included expert clinical assessment, in vivo confocal microscopy, histology on four primary corneal buttons and a corneal allograft with disease recurrence, immunohistochemical staining, TEM, and mutational analysis of *TGFBI.* The results of these investigations suggest a novel genotype-phenotype correlation. Comparison of these data with an extensive review of the literature supports our view that this reported dystrophy, although exhibiting some features of both CDB1 and CDB2, does not meet the diagnostic classification for either, thereby adding to the complexity of the pathogenesis of the *TGFBI* corneal dystrophies.

## Methods

### Patient recruitment

**Figure 1:**
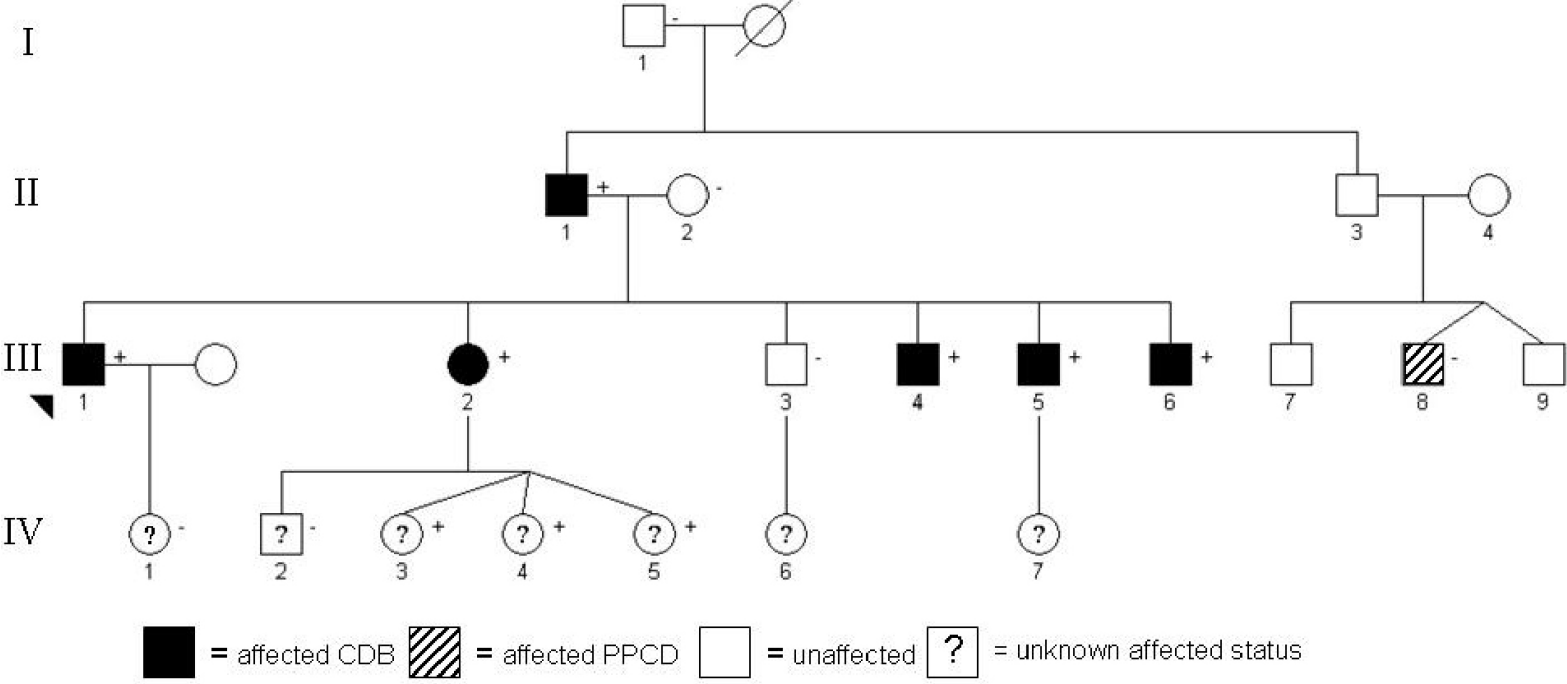
A four-generation pedigree illustrating affected and unaffected individuals. Either "+" or "–" denotes presence or absence of mutant allele, and denotes all the individuals examined. The three younger members of the pedigree (triplets) with the mutant allele were age 3 years at time of last examination, and appeared clinically unaffected. CDB=Bowman’s layer corneal dystrophy, PPCD=posterior polymorphous corneal dystrophy.

Individuals from a single four-generation pedigree of Caucasian ethnicity (Figure 1) with autosomal dominant CDB were recruited from the Department of Ophthalmology, Auckland, New Zealand District Health Board and then assessed at the University of Auckland Ophthalmology Clinic. The study design adhered to the tenets of the Declaration of Helsinki, with Institutional Research Ethics Board approval. After providing verbal and written explanations of the purpose and possible consequences of the study participants gave their informed consent.

### Clinical examination

All subjects underwent extensive clinical examination by a single, experienced, examiner including Snellen visual acuity, slit-lamp biomicroscopy, and anterior segment photography. Three affected individuals (II:1, III:1, and III:5) were also studied by laser scanning in vivo confocal microscopy (Heidelberg Retina Tomograph II, Rostock Corneal Module (RCM); Heidelberg Engineering GmbH, Heidelberg, Germany).

### Histopathology and transmission electron microscopy

The corneal tissue from four affected individuals - four primary corneal buttons (II:1, III:1, III:2, and III:5) and one allograft (II:1) - was examined histopathologically following penetrating keratoplasty (PKP) for reduced visual acuity. Tissue for light microscopy was fixed in 10% formalin in 0.1 M phosphate buffer then stained with hematoxylin and eosin (H&E), Congo red, periodic acid Schiff (PAS), alcian blue, and Masson’s trichrome. One of these patients had undergone repeat keratoplasty for recurrence of the dystrophy within the corneal graft and the tissue recovered following this was also studied by TEM. This tissue was fixed in Karnovsky’s fixative then washed in 0.1 M phosphate buffer. The tissue was post fixed in 0.5% osmium tetroxide in 0.1 M phosphate buffer for 2 h before being dehydrated through a graded ethanol series and embedded in procure 812 resin. Resin blocks were cut into sections and placed on copper grids for transmission electron microscopy.

### Immunofluorescence

One quarter of the tissue sample (II:1) was fixed in 2.5% paraformaldehyde for 1 h, then washed and mounted in TissueTek® OCT medium. Sections (20 μm) were cut in an antero-posterior direction and collected onto Superfrost Plus slides (Menzel Glaser, Germany). Standard immunofluorescence labeling techniques were used to incubate the sections with affinity-purified rabbit anti-TGFBI antibody [25] (Antibody courtesy of Dr Andrew Huang, University of Minnesota, Minneapolis, MN) overnight at 4 °C and a CY3 conjugated secondary antibody for 2 h. Sections were counterstained with DAPI, mounted in Citifluor medium (Citifluor Ltd, UK) and coverslip applied. Slides were viewed using a Leica DMRA microscope and images captured using a Nikon Digital Sight DS-U1 camera system and NIS Elements BR2.30 Software. Images were combined using Adobe Photoshop®.

### Mutational analysis

Biologic samples (peripheral blood, buccal swab, saliva) were collected from six members of the pedigree affected with CDB, one member affected with posterior polymorphous dystrophy (PPCD), three unaffected members, and five individuals of unknown affected status due to their age. Leukocyte DNA extraction was performed using the salt extraction method [26]. Buccal and saliva DNA were extracted following manufacturers guidelines (Purgene® DNA purification kit; Gentra Systems, Minneapolis, MN and Oragene; DNA Genotek, Ottawa, ON, Canada, respectively). PCR amplification of all the coding exons of *TGFBI* (exons 1–17) was undertaken using previously described primers and conditions [12]. Following column purification using a HighPure PCR purification kit (Roche Diagnostic, Mannheim, Germany), the product was sequenced directly according to protocols accompanying the ABI *BigDye* terminator kit v3.1. Bidirectional sequencing of amplicons was undertaken on an ABI 3100 prism genetic analyzer (Applied Biosystems Inc., Foster City, CA), to collect and analyze the sequence data. Nucleotide sequences were compared with the published *TGFBI* cDNA (cDNA) sequence (GenBank NM_000358).

The exon 14 sequence variant was confirmed with NlaIII restriction enzyme digest using 9 μl PCR product, 0.2ul NlaIII (New England BioLabs, Beverly, MA), 1.2 μl NEBuffer 4 (50 mmol potassium acetate, 20 mM Tris acetate, 10 mmol magnesium acetate, 1 mmol DDT, pH7.9 at 25 °C), 0.12 μl BSA (100X), and 1.48 μl H_2_0 (total 12 μl), which was incubated overnight at 37 °C.

### Paternity testing

Paternity testing was undertaken on individuals I:1, and II:1 using 12 heterogeneous polymorphic markers throughout the genome with known population allele frequencies, which were genotyped on the ABI prism. Parental index was calculated using Brenners formula. Fifty unaffected control individuals (100 control chromosomes) of matched ethnicity also underwent NlaIII restriction enzyme digestion of the exon 14 amplicon.

## Results

### Clinical presentation

Six members of the pedigree affected with CDB, one member affected with PPCD, three unaffected members, and five members of unknown affected status due to their age  were identified and examined. Affected members were diagnosed at a mean age of 10.3±1.5 (mean±SD) years with symptoms of painful recurrent corneal erosions. A rapid, progressive reduction in visual acuity was reported during their teenage years and at the time of review by the authors best corrected visual acuity (BCVA) varied significantly in affected eyes (6/18^−2^-6/60; Table 1).

**Table 1:** Summary of clinical presentation and results of investigations within the pedigree.

**Individual**	***TGFBI* genotype**	**Current clinical status (determined by symptoms/ slit lamp examination)**	**Sex**	**Current age (years)**	**Age at onset (years)**	**BCVA pre-treatment (PTK/PKP)**		**Tissue diagnosis**	**PTK**	**Age at corneal graft**		**Recurrence in graft**
						**OD**	**OS**			**OD**	**OS**	
I:1	Wt/wt	Not affected	M	95	-	-	-	-	-	-	-	-
II:1	wt/H626P	Affected	M	66	9	39623		Yes	No	61	44	Yes OU.
II:2	Wt/wt	Not affected	F	63	-	-	-	-	-	-	-	-
III:1	wt/H626P	Affected	M	35	12	17685	17685	Yes	No	35	34	No
III:2	wt/H626P	Affected	F	33	11	37428		Yes	OD	No	19	Trauma graft
III:3	Wt/wt	Not affected	M	32	-	-	-	-	-	-	-	-
III:4	wt/H626P	Affected	M	30	8	Not known	Not known	No	OU	No	No	N/A
III:5	wt/H626P	Affected	M	26	11	37063		Yes	OD	No	15	Failed graft
III:6	wt/H626P	Affected	M	20	11	37425	22068	No	OU	No	No	N/A
III:8	wt/wt	Affected (PPCD)	M	37	14	39605	39605	No	No	No	No	N/A
IV:1	wt/wt	Not affected	F	2	-	-	-	-	-	-	-	-
IV:2	wt/wt	Not affected	M	10	-	-	-	-	-	-	-	-
IV:3	wt/H626P	Not affected	F	3	-	-	-	-	-	-	-	-
IV:4	wt/H626P	Not affected	F	3	-	-	-	-	-	-	-	-
IV:5	wt/H626P	Not affected	F	3	-	-	-	-	-	-	-	-

Current clinical status refers to presence of symptoms and/or signs. OD=right eye, OS=left eye, OU=both eyes, wt=wild type, wt/H626p=individual with mutation, BCVA=best corrected visual acuity, PPCD=Posterior polymorphous corneal dystrophy, PTK=excimer laser phototherapeutic keratectomy, PKP=penetrating keratoplasty, M=male, F=female, N/A=not applicable. Information that is not provided about clinically unaffected individuals is marked with a dash (-).

**Figure 2:**
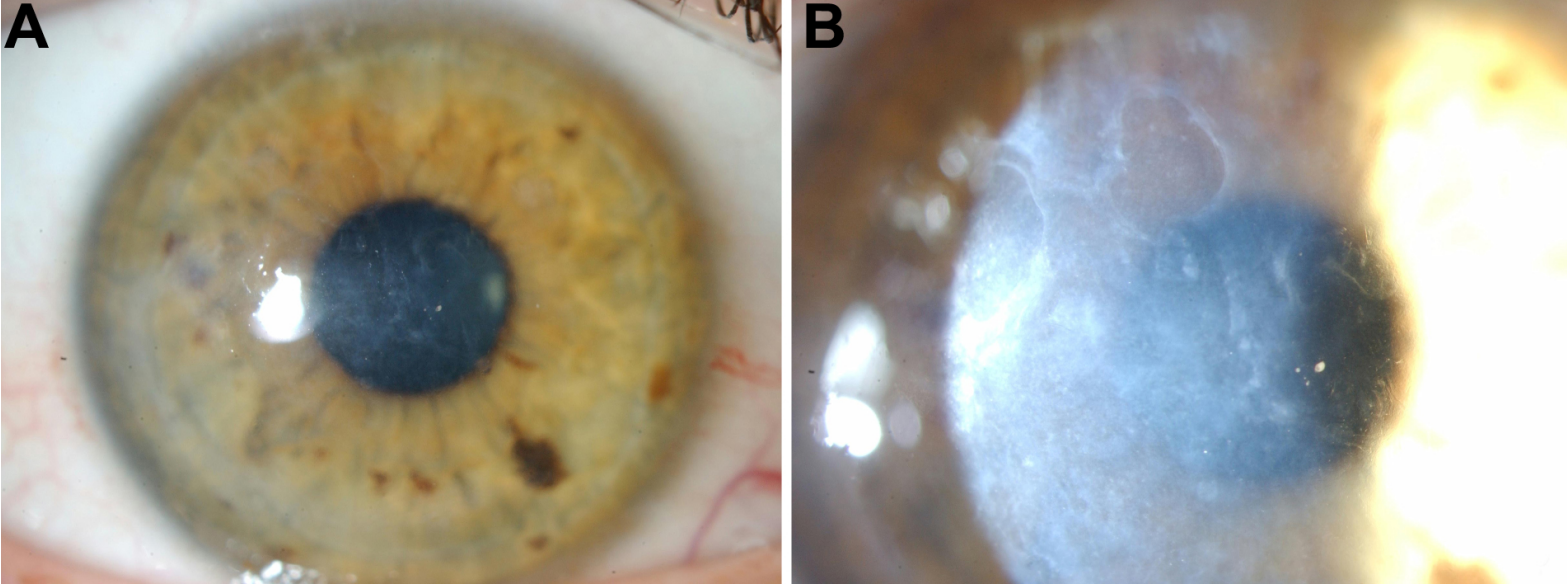
Representative slit-lamp biomicroscopy photographs illustrating corneal phenotype in an affected individual. **A**: 10X magnification diffuse illumination and **B**: 16X magnification broad slit lamp slit illumination of the left cornea of subject III:5.

Slit-lamp biomicroscopy typically showed an irregular corneal surface with discrete, gray-white opacities of various morphologies in the sub-epithelial area that projected anteriorly from the level of Bowman’s layer into the overlying epithelium. The overlying epithelium was intact. With longer duration from diagnosis these opacities were noted in increasing association with marked corneal scarring, neovascularization, and calcification. No stromal lattice lines were observed. Representative clinical photographs of the left cornea of an affected family member are shown in Figure 2. Corneal sensation was diminished on qualitative assessment in all affected eyes.

**Figure 3:**
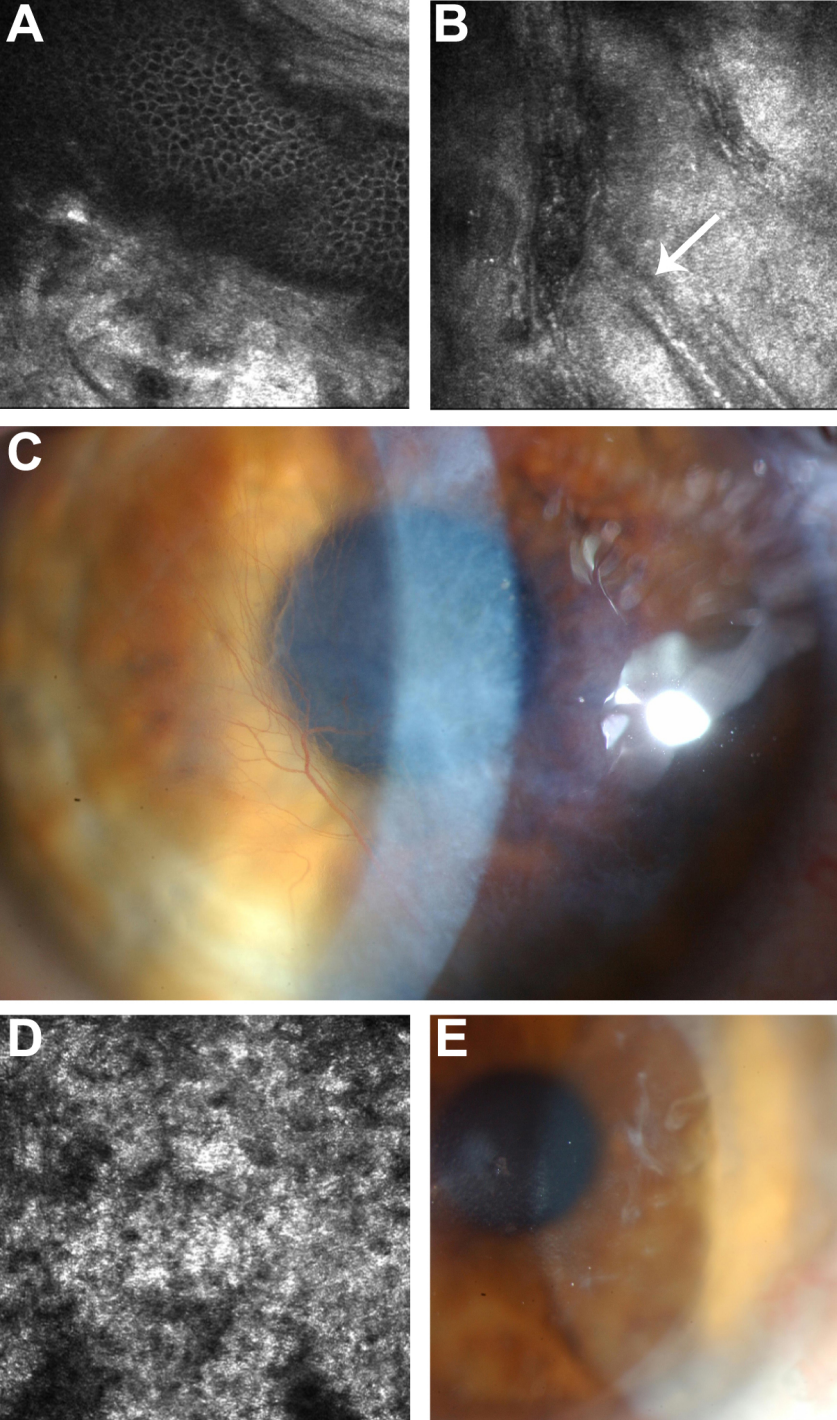
In vivo confocal microscopy images of representative affected family members.  The right eye (virgin cornea) of the proband (frame size 400 µm x 400 µm) at the level of (**A**) basal epithelial layer illustrating focal deposition of homogeneous, reflective materials with rounded edges, and hyporeflective borders and (**B**) posterior stroma showing extensive scarring with arrow indicating “ghost” blood vessels. **C**: Corresponding slit-lamp biomicroscopy photograph illustrating dense scarring. **D**: In vivo confocal microscopy of the allograft in the right eye of subject II:1 at the level of Bowman’s layer which has been completely replaced by diffuse, homogeneous, reflective material (frame size 400 µm x 400 µm). **E**: Corresponding slit-lamp biomicroscopy photograph illustrating recurrence of the dystrophy within the peripheral right corneal graft of subject II:1, 4 years following penetrating keratoplasty.

In vivo laser scanning confocal microscopy (IVCM) was performed on three of the affected subjects. Within the basal epithelial layer of the proband’s right eye, focal deposition of homogeneous reflective materials with rounded edges and hyporeflective borders was observed (Figure 3A). Extensive hyper-reflectivity extending throughout the full thickness of the stroma consistent with scarring, neovascularisation and ghost vessels was also noted (Figure 3B,C). This hyper-reflectivity in combination with the absence of focal, highly reflective, small granular materials at the same level has recently been described as a further feature of CDB2, thereby differentiating it from CDB1 [11]. Ghost vessels have well defined margins and have hyporeflective borders (as shown in Figure 3B). Also, blood vessels are clearly visible clinically (Figure 3C) and correlate with the appearance and location of structures observed in Figure 3B. Amyloid deposits appear as hyperreflective, linear, and branching structures with varying reflectivity and poorly demarcated margins. Such structures were not present on any IVCM images from the subject.

In the right eye of subject II:1, which had undergone an allograft four years earlier, diffuse, homogeneous, reflectivity was noted at the level of Bowman’s layer (Figure 3D) which correlated with the clinical appearance of recurrence of the dystrophy on slit-lamp biomicroscopy (Figure 3E).

Four affected individuals underwent PKP (six eyes), while four underwent excimer laser phototherapeutic keratectomy (PTK; six eyes) that produced significant improvements in both their symptoms and visual acuity. One subject (II:1) underwent repeat PKP for recurrence of the dystrophy 18 years after the initial corneal graft in the left eye. The clinical features of the pedigree are summarized in Table 1.

### Histopathologic analysis

All four corneal buttons from the primary grafts demonstrated subepithelial deposits of hyaline material of variable thickness that did not stain red with Masson trichrome and was not present within the anterior stroma per se. Bowman’s layer was absent/replaced over much of the excised cornea with poor basal lamina formation noted, and areas of poor adhesion of the basal cells to Bowman’s layer (where present) and the hyaline material where Bowman’s layer was absent. The deposits of hyaline material were PAS, Alcian blue, and Congo red negative. In two of the corneas there were extensive secondary changes of neovascularization, calcification, and scarring. There were no other deposits seen within the stroma, and specifically no amyloid staining. Descemet’s membrane and the endothelium were normal in all corneas.

**Figure 4:**
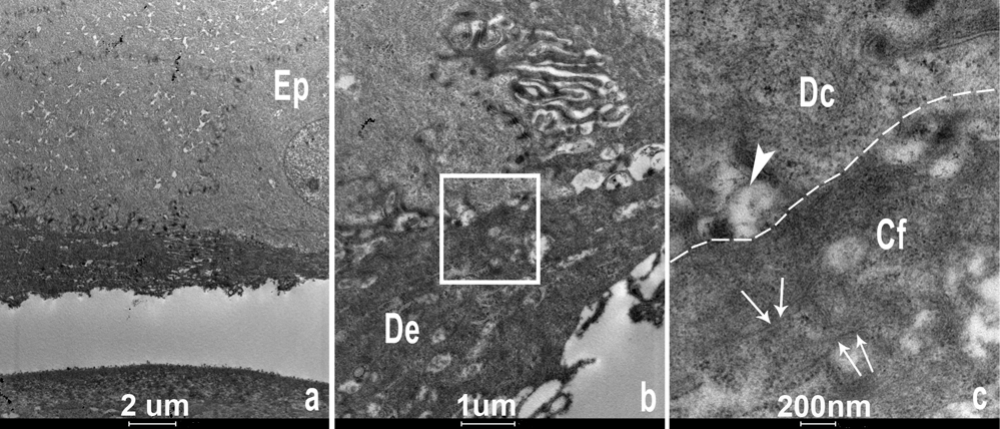
Transmission electron microscopy images of the corneal allograft from subject II:1. **A**: Low magnification photomicrograph showing basal epithelial cells (Ep) overlying a band of deposit (De), at higher magnification in **B**. The insert in **B** is shown in higher magnification in **C**, demonstrating the degenerating basal epithelial cell (Dc) above the dotted line, and below the subepithelial deposit, consisting of irregular aggregates of fibrils (Cf) as demonstrated with arrows. The large arrowhead shows a degenerating adhesion body.

**Figure 5:**
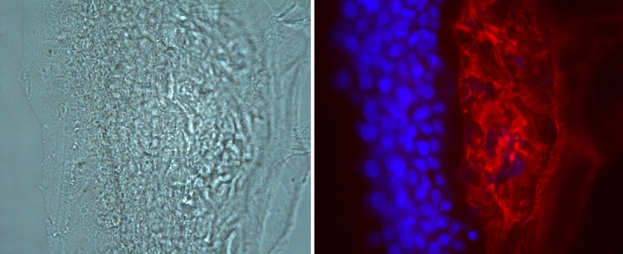
Immunohistochemical staining of a corneal allograft specimen (II.1).  The presence of TGFBI associated protein is demonstrated in the sub epithelial deposit (red). Corneal epithelial nuclei are stained with DAPI (blue). The right hand image shows the corresponding phase contrast light microscopy image. Magnification 40X.

Light microscopy of the corneal allograft from II:1, showed similar histopathological features. TEM examination of this corneal specimen showed an irregular epithelium with poor attachment to the underlying substratum (Figure 4). Although this could be an artifact of tissue processing, this was consistent with the clinical findings of a loose superficial layer that could easily be peeled off underlying corneal tissue. On TEM a band of subepithelial material largely obliterated Bowman’s layer. This appeared to be composed of irregular aggregates of curved fibrils intermingled with collagen fibers. No rod shaped bodies were identified. Immunohistofluorescence confirmed the presence of TGFBIp within this subepithelial deposit (Figure 5).

### Molecular genetic analysis

**Figure 6:**
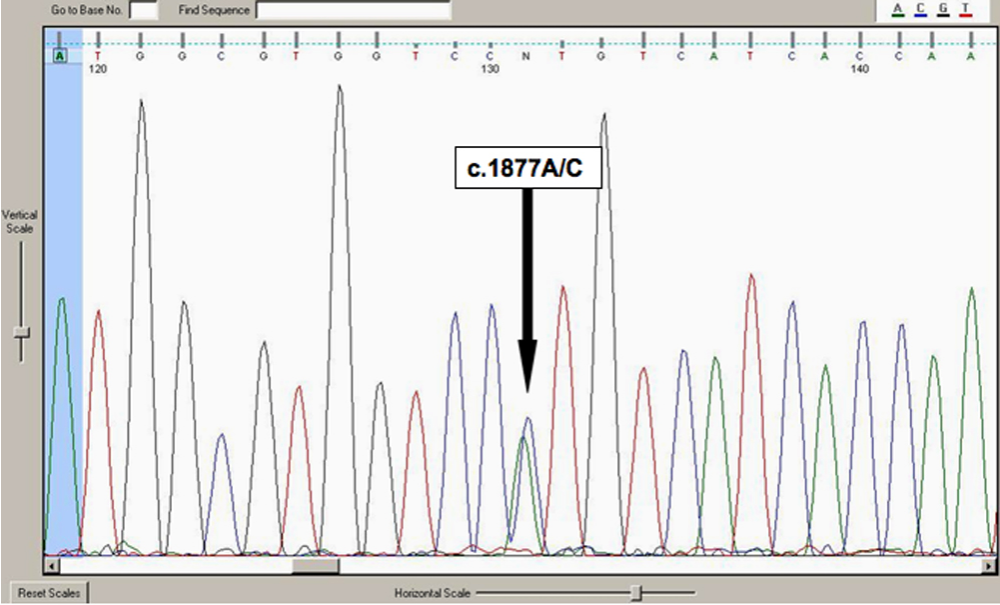
Electropherogram of TGFBI exon 14 in an affected individual.  This revealed a missense transversion A→C at nucleotide 1877, resulting in a substitution of histidine by proline (CAT→CCT, H626P).

Bidirectional DNA sequence analysis revealed a missense transversion A→C at nucleotide 1877 in exon 14 (c.1877A>C), resulting in a substitution of histidine by proline (CAT→CCT, p.H626P; Figure 6). In addition the proband was heterozygous for one previously described SNP in exon 6, (c.651C>G, L217L). Due to the removal of an NlaIII restriction site, the presence of the mutation c.1877A>C could be confirmed by electrophoresis after enzymatic digest of exon 14 in affected but not unaffected family members, or unaffected, unrelated, individuals. The mutation was confirmed by sequencing after enzyme digestion in all affected individuals, co-segregated with disease in the clinically affected individuals in this pedigree, and was also present in three younger members of the pedigree (IV:3, IV:4, and IV:5). As these triplets were three years of age at the time of the examination, affected status could not be determined, therefore segregation with these individuals, and with individual IV:1 (age 2) cannot be commented on. The cousin with PPCD (III:8) also did not carry the c.1877A>C sequence change. H626P was not present in 100 control chromosomes.

A parental index of >99.5% is highly suggestive of paternity for individuals I.1 and II.1 (data not shown). Maternity testing was unable to be performed as I:2 is deceased, but review of her eye notes before her death record no evidence of corneal abnormality at the age of 78 years.

## Discussion

Phenotypically this pedigree had some features that overlap with both CDB1 and CDB2. Features typically associated with CDB1 include marked visual loss (BCVA range pre-treatment 6/18^−2^-6/60), a young age at presentation (10.3±1.5 years, mean± SD) and early extensive recurrence observed following PKP in one individual [5]. Slit-lamp biomicroscopy also revealed confluent opacities that appeared geographic in shape as opposed to the honeycomb shaped opacities of CDB2. However, histopathology revealed that the subepithelial deposits did not stain red with Masson trichrome - a feature more characteristic of CDB2, often reported as minimal or questionable staining [5,14,27]. In vivo confocal microscopy images were consistent with reported findings in CDB2 [11]. Of the 5 youngest members of the pedigree examined and tested, three carried the mutation but were apparently clinically unaffected on slit lamp examination. However, they were at an age (3 years) much younger than the mean onset of disease symptoms/signs (10.3±1.5) in this pedigree.

Unfortunately tissue samples were not available on all subjects treated for corneal opacification, decreased vision and recurrent erosions. Although subjects with severe scarring and vascularization (and those treated before 1990) were treated by PKP, in selected members of the pedigree with the primarily superficial corneal lesions seen early in this dystrophy, excimer laser phototherapeutic keratectomy (PTK) was used successfully [28]. Having been shown to be a safe and effective alternative to re-grafting it has recently been used by the authors to treat the recurrence of the dystrophy in subject II:1 OD, with a promising early post operative result [29].

**Figure 7:**
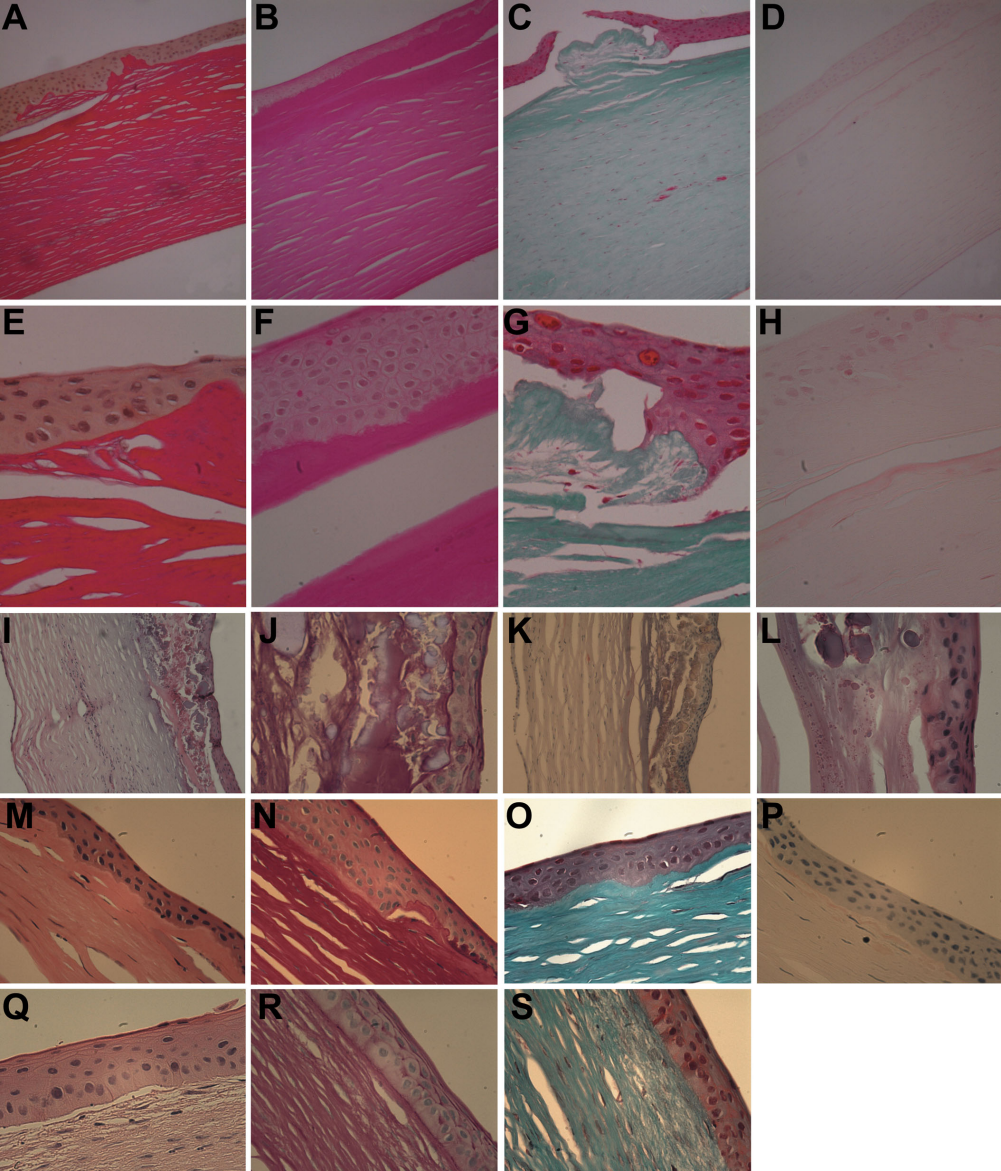
Representative light microscopy images of corneal buttons obtained at penetrating keratoplasty from affected family members.  An irregular epithelium is demonstrated with a variable thick band of hyaline avascular connective tissue and/or fibrocellular tissue below; Bowman’s layer is largely obliterated. This material did not stain red with Masson’s trichrome stain. (**A**-**H**: Individual III:1); **A**: AB-10X, **B**: PAS-10X, **C**: MTC-10X, **D**: CR-10X, **E**: AB-40X, **F**: PAS-40X, **G**: MTC-40X, **H**: CR-40X. (**I**-**L**: Individual II:1), **I**: HE-5X, **J**: PAS-40X, **K**: HE-40X, **L**: CR-10X. (**M**-**P**: Individual III:2), **M**: HE-40X, **N**: PAS-40X, **O**: MTC-40X, **P**: CR-40X, and (**Q**-**S**: Individual III:5), **Q**: HE-40X, **R**: PAS-40X, **S**: MTC-40X. Abbreviations used in the legend are: AB=Alcian Blue, PAS=periodic acid Schiff, MTC=Massons trichrome, CR=Congo Red, and HE=hematoxylin and eosin.

On light microscopy, a variable thick band of hyaline avascular connective tissue and/or fibrocellular tissue was identified beneath the corneal epithelium which did not stain red with Masson’s trichrome stain. (Figure 7) In the allograft specimen, the characteristic rod-shaped bodies observed in CDB1 were clearly not identified on TEM, and high magnification TEM demonstrated the subepithelial deposit immediately underlying degenerating basal epithelial cells. This deposit appears to consist of cell debris with some irregular aggregates, and scattered fibrils. These histopathological observations are not classically characteristic of either CDB1 or CDB2, although perhaps the presence of fibrils is more in keeping with a CDB2 variant [5,14,19]. Immunofluorescence demonstrated TGFBIp within the subepithelial deposit, which is consistent with the findings of Streeten et al. [27], who showed strong specificity of the stain to the curly fibers. However TGFBIp has been demonstrated in secondary corneal amyloidosis (confirmed with Congo red) in non-*TGFBI*-associated disease [24]. No Congo red staining was observed in any of our 4 corneal specimens, therefore this subepithelial band is unlikely to represent an amyloid deposit, however, the presence of TGFBIp is apparently not sufficient to differentiate a dystrophic deposit from scar tissue [24].

The novel association of the mutation H626P with this atypical CDB phenotype provides further evidence of the complexities of the classification and nomenclature of the *TGFBI* corneal dystrophies. This genotype-phenotype correlation is unique from two perspectives: First -historically CDB has predominantly been associated with 2 different distinct mutations; R124L with CDB1, and R555Q with CDB2 [13]. Second, the H626P mutation has been reported with a significantly different phenotype - late onset, asymmetric, lattice, dystrophy, (variant LCD, classified by the authors as CDLI/IIIA) [12], characterized by a dense haze associated with lattice lines, demonstrating multiple fusiform amyloid deposits in the stroma of an allograft corneal button. Recently the H626P change was shown to segregate with a superficial dystrophy that also showed the presence of amyloid [30]. A further mutation at this same base pair c.1877A>G resulting in H626R has also been shown to co-segregate with a late onset, asymmetric, form of lattice dystrophy (with previous numbering c.1924) [12,19,31-34]. One family had disease onset at 27 years of age, no recurrent erosions, and PKP necessary in the fifth decade [33]. H626R is postulated as being responsible for up to 75% of LCD seen in a Vietnamese population [32], termed LCDIIIB, and distinctive in that it has a later age of onset, asymmetry is common, and the lattice lines are thicker and more rope-like. Recently this mutation is described in a family with granular corneal dystrophy (GCD) [35].

In a recent paper the phenotype associated with the R124L mutation was well characterized, but as a corneal biopsy in the proband failed to distinguish between CDB1 and CDB2, the authors expound the virtues of genetic testing to determine the diagnosis [36]. These genotype-phenotype correlations (CDB1–R124L and CDBII–R55Q) have been confirmed numerous times with more detailed clinical characterization [13,14,36-38], and a suggested nomenclature for these dystrophies is “Classic” CDB [4].

In contrast CDB occurring with mutations other than the 124 or 555 mutations can be termed “Atypical” CDB; however histology is not recorded in these cases. Rozzo et al. [39] described F540Δ in CDB1, but reclassified the phenotype as late-onset lattice IIIa (CDL3A). Two pedigrees are described with G623D [40,41], one of which was CDB1 with apparent non-penetrance [40], and the other had deposits in Bowman’s layer, anterior stromal lattice lines, with symptomatic onset in the fourth decade [41].

Paternity testing is highly suggestive of a de novo mutation in *TGFBI* in II.1, by confirming the paternity of his unaffected father, however maternity could not be confirmed as his reportedly unaffected mother had died. Further possibilities which have not been investigated further include parental germ line mosaicism, or non-penetrance from the reportedly unaffected mother, although none of her other relatives were examined. Certainly de novo mutations have been documented in TGFBI-associated disease, as well as germ-line mosaicism [36,42,43]. It would appear that *TGFBI* does have some mutational “hot-spots,” where identical random mutations occur at an increased frequency at the same site of a gene. Korvatska et al. [44,45] demonstrated different haplotypes segregating with R124L in Japanese families, and in different ethnicities. They suggested that mutations at codons 124 and 555 in particular represent multiple independent occurrences.

With the addition of this report, significant phenotypic variability has now been demonstrated with the histidine at position 626, segregating with CDB, GCD, and LCD IIIB phenotypes. Likewise mutations within close proximity of the 626 residue also appear to show phenotypic variability. Previous modeling of the structure of TGFBIp demonstrates that His626 makes a crucial stabilizing hydrogen bond within the Fas4 domain [17]. This hydrogen link would be abolished by replacement of a proline, and therefore is likely to severely destabilize the domain [17]. So why is there such broad phenotypic variability seen in exon 14 mutations, and of specific relevance here, to mutations involving the His 626? Clout et al. [17]. also modeled His626Arg, which as well as abolishing the hydrogen bond, is likely to make the protein very unlikely to fold because of steric hindrance by the arginine residue

Although Fas4 domain mutations are thought to result in misfolding of the protein, a major role of the Fas domain is to mediate cell adhesion. TGFBIp also polymerizes to form a fibrillar structure and strongly interacts with type I collagen, laminin, and fibronectin [46]. It is unclear how the H626P change results in the subepithelial deposit observed with poor adherence to underlying tissues in this family, however one possibility is the accumulation of mutant misfolded protein is somehow deleterious to the cell, and a further theory is that the mutant TGFBIp cannot participate adequately in its usual binding functions, including polymerization to self and/or to other tissues [17,46,47]. There does however appear to be a corneal specific factor which influences this process, as other epithelial tissues in the body from individuals with a mutation, do not appear to manifest with TGFBIp deposits [47].

Other modifying factors could include co-existent SNPs, although there is no prior evidence that the L217L SNP present in exon 6 of the proband has any functional role. Interestingly, within this pedigree, slit-lamp biomicroscopy of the proband’s cousin (III:8) clinically revealed PPCD. He did not carry the mutation H626P, which could suggest that a mutation in a second gene may be responsible for his phenotype, and perhaps even modify the expression of the H626P mutation in this family.

The association of this pedigree with the phenotype CDB and the mutation H626P is in keeping with the majority of the published *TGFBI* mutations occurring either at Arg124 or within the Fas4 domain, and the absence of this mutation in our 100 control chromosomes and in previous controls suggests it is pathogenic rather than polymorphic. This novel genotype-phenotype correlation challenges our current knowledge of the “phenotypic specificity” that CDB1 segregates with the R124L genotype, and CDB2 with R555Q. Likewise mutations at the 626 residue segregate with non-lattice phenotypes. To explain the marked difference in phenotype between this pedigree and those described with H626 mutations, it is possible that a modifying genetic or environmental mechanism may be involved, the nature of which is unknown. Certainly further investigation is warranted on the nature of the deposits and the protein’s properties. This H626P CDB phenotype does not fit neatly into either CDB1 or CDB2 categories, and demonstrates the striking phenotypic variability observed with *TGFBI* mutations. This study and recent molecular evidence therefore highlights the ongoing complexity of the pathogenesis of *TGFBI* associated corneal dystrophies, and emphasizes the need to review the current nomenclature used to define *TGFBI*-associated dystrophies of Bowman’s layer.
